# A new genus (Copepoda, Harpacticoida, Laophontidae) from Jeju Island of Korea

**DOI:** 10.3897/zookeys.447.7603

**Published:** 2014-10-16

**Authors:** Jinwook Back, Wonchoel Lee

**Affiliations:** 1 Marine Biological Resources Division Planning Office, Marine Biodiversity Institute of Korea, Seocheon, 427–100, Korea; 2 Department of Life Science, College of Natural Sciences, Hanyang University, Seoul 133–791, Korea

**Keywords:** *Jejulaophonte*, Taxonomy, DNA barcode, intertidal, Marine

## Abstract

A survey on the harpacticoid copepods from an intertidal zone in Hyeopjae sandy beach, Jeju Island, Korea, resulted in the discovery of an unusual laophontid, *Jejulaophonte hyeopjaeensis* **sp. n.**, which cannot be placed in any extant genus within the family. To accommodate the species, a new genus of the family Laophontidae T. Scott, 1905 is proposed and fully described here. The new species is closely related to the lineage of the five primitive genera, *Carraroenia* McCormack, 2006, *Coullia* Hamond, 1973, *Hemilaophonte* Jakubisiak 1933, *Psammoplatypus* Lee & Huys, 1999, and *Robustunguis* Fiers, 1992 (the CCHPR-lineage) by the reduced P2 endopod, ovate shape of the female P5 exopod and sexual dimorphism in the P3 endopod. However, it displays discrepancies from the species of the CCHPR-lineage in the presence of an inner seta on P3 and P4 exp-2, four setae on P4 enp-2, and an inner seta on P3 and P4 enp-2 in the female. Furthermore, no other species within the family Laophontidae has three setae on P2 exp-3 and a seta on P2 enp-2 at the same time. The new species has sexual dimorphism in the antennule, genital segmentation and the legs from P2 to P5. The terminal seta on the second endopodal segment of P2 in the male is longer than that in the female. The endopod of P3 is 3-segmented and displays a short inner apophysis on the second segment in the male. The outer setae on the exopod of P3 and P4 are distinctly thicker and stronger in the male than in the female. Mitochondrial cytochrome oxidase subunit I (mtCOI) sequencing of the new species has been realized in order to be used in future phylogenetic analysis.

## Introduction

Laophontid harpacticoids inhabit various environments including deep sea (Willen 1996; Lee and Huys 1999; Huys and Lee 2000). However the family Laophontidae is found mainly clinging to epiphytal habitats (Boxshall and Halsey 2004). Furthermore, several genera have been found in association with various organisms, for example, *Hemilaophonte janinae* Jakubisiak, 1933, collected from washings of the spider crab, *Maia squinado* (Herbst, 1788). They have highly reduced and modified appendages as specific adaptations to their host.

The family Laophontidae T. Scott, 1905 is a large group of harpacticoid copepods, comprising over 262 species in 63 genera and two families: Esolinae and Laophontinae (Boxshall and Halsey 2004). The type species, *Laophonte cornuta*, was published by Philippi (1840) and T. Scott (1905) proposed the family name, however he did not define the characters of this taxon. Lang (1944) divided the family in three subfamilies: Laophontinae, Normanellinae, and Donsiellinae. Later, Huys and Willems (1989) upgraded the Normanellinae to family rank and Hicks (1988) revised the Donsiellinae, creating four new genera and removing it to the family Thalestridae. Although many genera and species have been moved to other families, because of the numerous new genera and species described since Lang’s (1965) key there is no easy way to identify the genera of Laophontidae. Huys and Lee (2000) provided a key to genera of subfamily Esolinae which included eight genera, and Huys (2009) suggested a key to species of five genera having reduced P2 endopod.

In this study, a survey on the harpacticoid copepods from an intertidal zone in Jeju Island, Korea resulted in the discovery of an unusual laophontid, which could not be allocated to any extant genera in the family Laophontidae. Sandy sediments around Jeju Island originate from volcanic rock called basalt. The sediment type of studied area, Hyeopjae beach, is silvery sand that is mixed with sand and various shell dusts. In addition, there are a lot of marine algae that are washed ashore by waves. The family Laophontidae includes various organisms, which are adapted to a habitat style, namely their cylindrical body shape and a reduced segmentation of their swimming legs (Gheerardyn et al. 2007). These interstitial species including associates with other invertebrates or alga also present a reduced segmentation of the thoracic appendages. To accommodate the new species, a new genus of Laophontidae is proposed and fully described here. In addition the mitochondrial cytochrome oxidase subunit I (mtCOI) sequences are obtained for using as molecular barcode of the new species.

## Materials and methods

Sediments were collected by a small shovel and acryl cores (diameter 5.4 cm) in a submerged area of Hyeopjae sandy beach, Jeju island, Korea (about 1 m depth). The sediment samples were fixed in 5% neutralized formalin for taxonomic study. Copepods are extracted from the sediment samples by using the Ludox method (Burgess 2001) and fixed in 70% ethanol. Specimens were dissected in lactic acid, and the dissected parts were mounted on slides in lactophenol mounting medium. Preparations were sealed with transparent nail varnish. All drawings have been prepared using a camera lucida on an Olympus BX51 differential interference contrast microscope.

For scanning electron microscopy copepods were prefixed in 70% ethanol, dehydrated through graded ethanol for Hitachi S-2380N in Hanyang University or acetone series for Philips XL-30 in the Natural History Museum London, critical point dried, mounted on stubs using double-sided tape, coated with gold, and then examined with a scanning electron microscope (Hitachi S-2380N, Philips XL-30).

The descriptive terminology is adopted from Huys et al. (1996). Abbreviations used in the text are: A1, antennule; A2, antenna; ae, aesthetasc; exp, exopod; enp, endopod; P1–P6, first to sixth thoracopod; exp(enp)-1(2, 3) to denote the proximal (middle, distal) segment of a three-segmented ramus. Specimens are deposited in the National Marine Biodiversity Institute of Korea. Scale bars in figures are indicated in µm.

For DNA sequencing, copepods were collected using hand net (mesh size 63 µm). Salt was washed from these samples on the sieve (mesh size 38 µm) and then, the samples were fixed in pure (100%) ethanol. Mitochondrial cytochrome oxidase subunit I (mtCOI) was amplified by polymerase chain reaction (PCR) using PCR premix (BiONEER Co). The amplification primers used were LCO-1490 (5´-GGT CAA CAA ATC ATA AAG ATA TTG G-3´) and HCO-2198 (5´-TAA ACT TCA GGG TGA CCA AAA AAT CA-3´) (Folmer et al. 1994) and premix (BiONEER). The amplification protocol was: 94 °C (1 min), 46 °C (2 min), 72 °C (3 min) carried out for 40 cycles. PCR products was purified with LAboPass PCR purification Kit (COSMO co, Ltd., Korea) and sequenced in both directions using an ABI 3730XL (COSMO co, Ltd., Korea).

## Systematics

### Order Harpacticoida Sars, 1903 Family Laophontidae T. Scott, 1904

#### Jejulaophonte gen. n.

Taxon classificationAnimaliaHarpacticoidaLaophontidae

http://zoobank.org/8526EE96-00AC-49D3-AB2C-D6969CF1D4E9

##### Diagnosis.

Laophontidae. Body elongate, sub-cylindrical, not dorsoventrally depressed; genital field with 2 setae each on P6 and small copulatory pore located in median depression; anal operculum well developed. Sexual dimorphism in antennules, P3–P6, and genital segmentation; rostrum small, fused at base; antennule with a small process in segment 2 and 7–segmented subchirocer in male, aesthetasc on segment 4 and 6 in female, 5 and 7 in male; maxillary syncoxa with 2 endites, endopod represented by 2 setae fused basally and 1 small naked seta; P1 exopod–2 with 5 setae; P2 smaller than P3 and P4; P3 enp-2 in male produced into a conspicuous apophysis.

##### Type and only species.

*Jejulaophonte hyeopjaeensis* sp. n.

#### Jejulaophonte hyeopjaeensis sp. n.

Taxon classificationAnimaliaHarpacticoidaLaophontidae

http://zoobank.org/ED9189D2-8C1A-4321-8CDC-65E5E117013E

Figs 1 , 2 , 3 , 4 , 5 , 6 , 7 , 8

##### Type locality.

Intertidal zone at Hyeopjae beach Jeju island, Korea (33°23'41"N, 126°14'22"E) on 10 April 2004 (type specimen). For DNA analysis, specimens collected on 3 June 2010 (for DNA analysis) at type locality.

##### Material examined.

Holotype 1♀ (CR235161) dissected on 9 slides. Paratypes 1♂ (CR235162) dissected on 8 slides, and 11♀♀ (CR235163), 5♂♂ (CR235164) in 70% alcohol. 9 specimens (6♀♀, 3♂♂) dried, mounted on stubs, and coated with gold for SEM. All specimens are from the type locality.

##### Etymology.

Specific name refers the type locality of new species, Hyeopjae beach, Jeju Island, Korea.

##### DNA-barcode

**(mt COI).** Sequences and traces were submitted to GenBank (GenBank Accession numbers: KF857218, KF857219)

##### Description of female.

**Figure 1.**
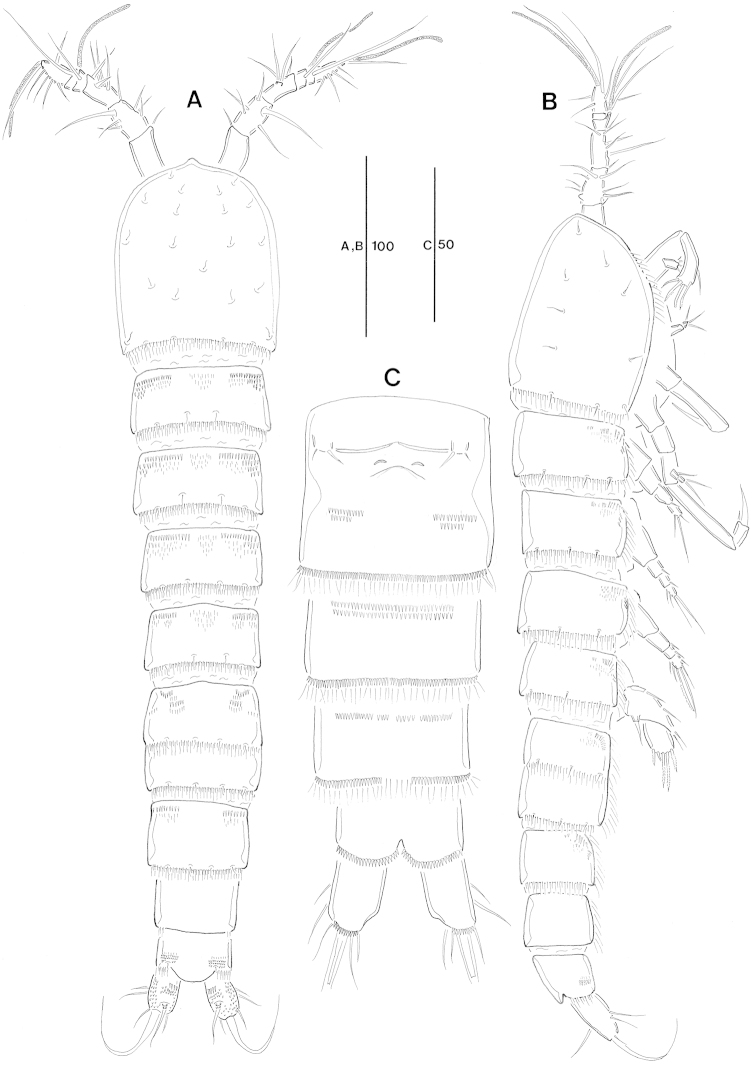
*Jejulaophonte hyeopjaeensis* gen. n., sp. n. (♀). **A** habitus, dorsal **B** habitus, lateral **C** urosome, ventral.

Total body (Fig. 1A, B) length from anterior margin of rostrum to posterior margin of caudal rami 477 µm (n = 6, mean = 472 µm). Maximum width 88 µm measured at midway of cephalothorax.

Body (Fig. 1A, B). Cylindrical and not dorsoventrally depressed with minute sensilla dorsally. Small sensilla well developed on the distal margin of prosomites and urosomites.

Rostrum (Fig. 1A). Diminutive, fused with cephalothorax, no sensilla.

Prosome (Fig. 1A). 4-segmented, comprising cephalothorax (bearing first pedigerous somite) and three free pedigerous somites. Cephalothorax subrectangular, wider than free somites. Cuticula between cephalothorax and first free somite distinctly pursed. Pleural areas of cephalic shield narrow and posterolateral angles rounded. Posterior margin of cephalothorax and all pedigerous somites with a row of long setules dorsally and laterally (Figs 1A, B, 8A). Free prosomites with spinules tuft on dorso-anterior surface and several setules on dorso-lateral margin.

**Figure 2.**
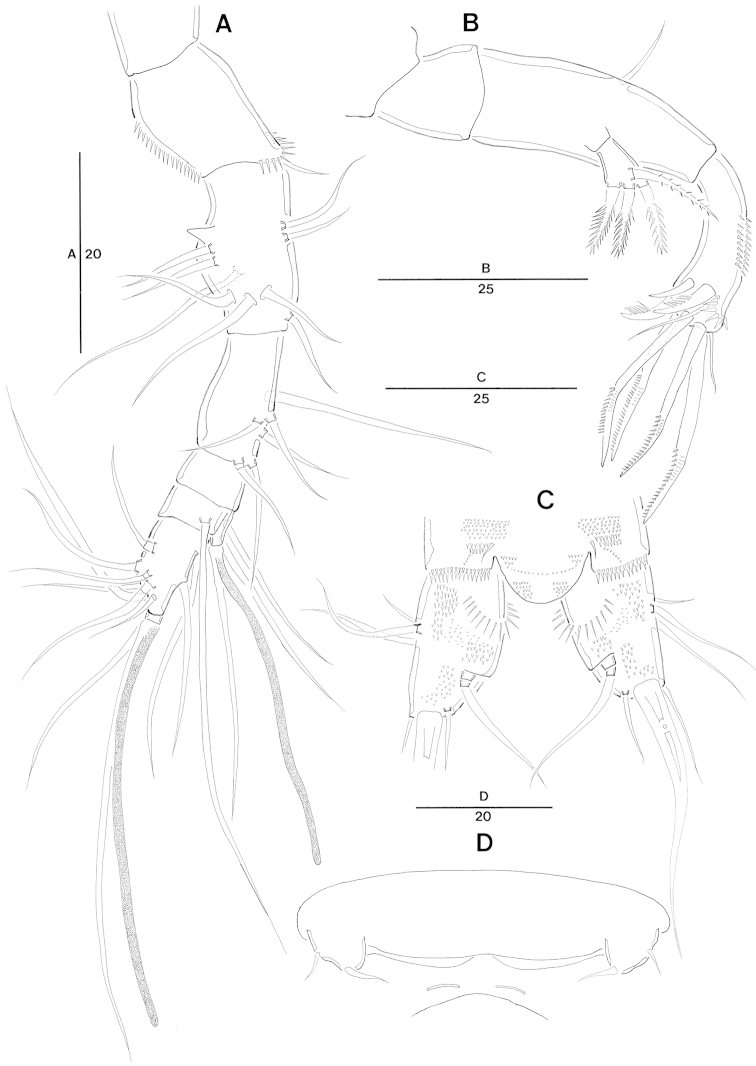
*Jejulaophonte hyeopjaeensis* gen. n., sp. n. (♀). **A** antennule, dorsal **B** antenna **C** anal somite and caudal rami, dorsal **D** genitial area with P6.

Urosome (Fig. 1A–C). 5-segmented, comprising P5-bearing somite, genital double-somite, and three free abdominal somites. Genital double-somite wide and original segmentation marked by a row of long setules and short spinules row arising from transverse surface ridge dorsally and laterally. Ventral surface bearing spinular tufts laterally. Each P6 (Fig. 2D) well developed opercula closing off paired genital apertures presented by one fig fused in middle, with 2 setae. Genital field (Figs 1C, 2D) located near the upper part of genital double-somite. Penultimate and anal somites distinctly narrow. Penultimate somite without sensilla dorsally. Anal somite with spinular tufts laterally; with well developed and smooth anal operculum (Figs 2C, 8H).

Caudal rami (Fig. 2C). Parallel, widely separated, dorsal surface with small minute spinules, and proximal inner margin with a lateral row of stout spinules. Each ramus with 7 setae: seta I smallest, setae II and III well developed and naked, seta IV naked, seta V longest and strongest, seta VI bare at the inner distal corner, seta VII naked and triarticulate at base.

Antennule (Fig. 2A). Slender, 6-segmented. Segment-2 with 1 small blunt process and 9 bare setae. Segment-4 carrying sub-cylindrical process furnished with 2 bare setae and 1 slender seta fused basally with aesthetasc. Apical acrothek consisting of an aesthetasc fused basally to 2 slender naked setae. Armature formula: 1–[1 bare], 2–[9 bare], 3–[6 bare], 4–[2 bare + (1+ae)], 5–[1 bare], 6–[9 bare + acrothek].

Antenna (Figs 2B, 8F). 3-segmented, comprising coxa, allobasis, and free 1-segmented endopod. Coxa small and bare. Allobasis with 1 bare abexopodal seta located about halfway along the segment. Exopod 1-segmented with 4 pinnate setae. Endopod, subtriangular pyramid-shaped, shorter than allobasis, spinule tuft on medial surface and with lateral armature consisting of 3 spines, 2 bare and 4 geniculate setae.

Labrum (Fig. 8B) with spinular ornamentation around distal margin; dense pattern of fine spinules anteriorly and distal patch of overlapping scales.

**Figure 3.**
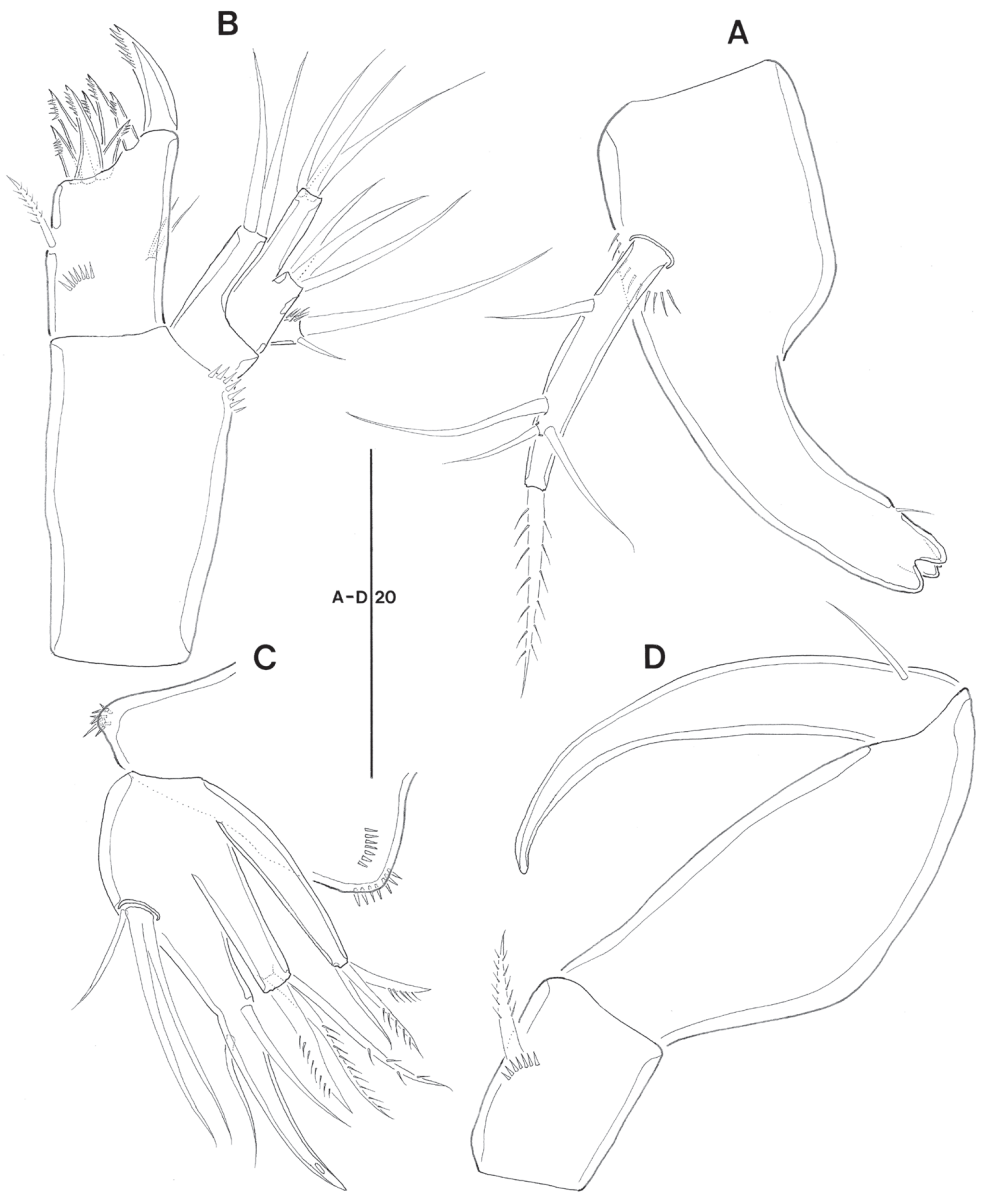
*Jejulaophonte hyeopjaeensis* gen. n., sp. n. (♀). **A** mandible **B** maxillule **C** maxiila **D** maxilliped.

Mandible (Fig. 3A). Small gnathobase armed with 1 slender bare seta on dorsal side and several blunt teeth. Mandibular palp probably representing fused basis and endopod; with 1 lateral (basal) bare seta, 3 sub-distal bare setae, and 1 distal pinnate seta.

Maxillule (Figs 3B, 8B). Praecoxa trapezoidal shape armed with a few spinules around outer margin; Arthrite well-developed with 2 juxtaposed setae near halfway on anterior surface, 1 plumose seta laterally, and 8 elements around distal margin. Coxa bearing cylindrical endite with 2 bare setae. Basal endite with 3 distal naked setae. Exopod 1-segmented, armed with 1 distal naked seta and 1 short bared seta. Endopod incorporated into basis, forming small peduncle with 3 naked setae.

Maxilla (Figs 3C, 8B). Syncoxa without spinules on surface and armed with 2 slender endites. Proximal and distal endites armed with 1 spine and 2 setae. Allobasis produced into a strong curved claw; accessory armature consisting of 2 naked setae proximally and 1 pore distally. Endopod incorporated into allobasis, consisting of 2 bare setae fused basally and 1 small naked seta.

Maxilliped (Fig. 3D). 3-segmented. Syncoxa with 1 plumose seta. Basis elongate without ornamentation. Enodpod drawn out as a smooth claw with 1 accessory naked seta anteriorly.

**Figure 4.**
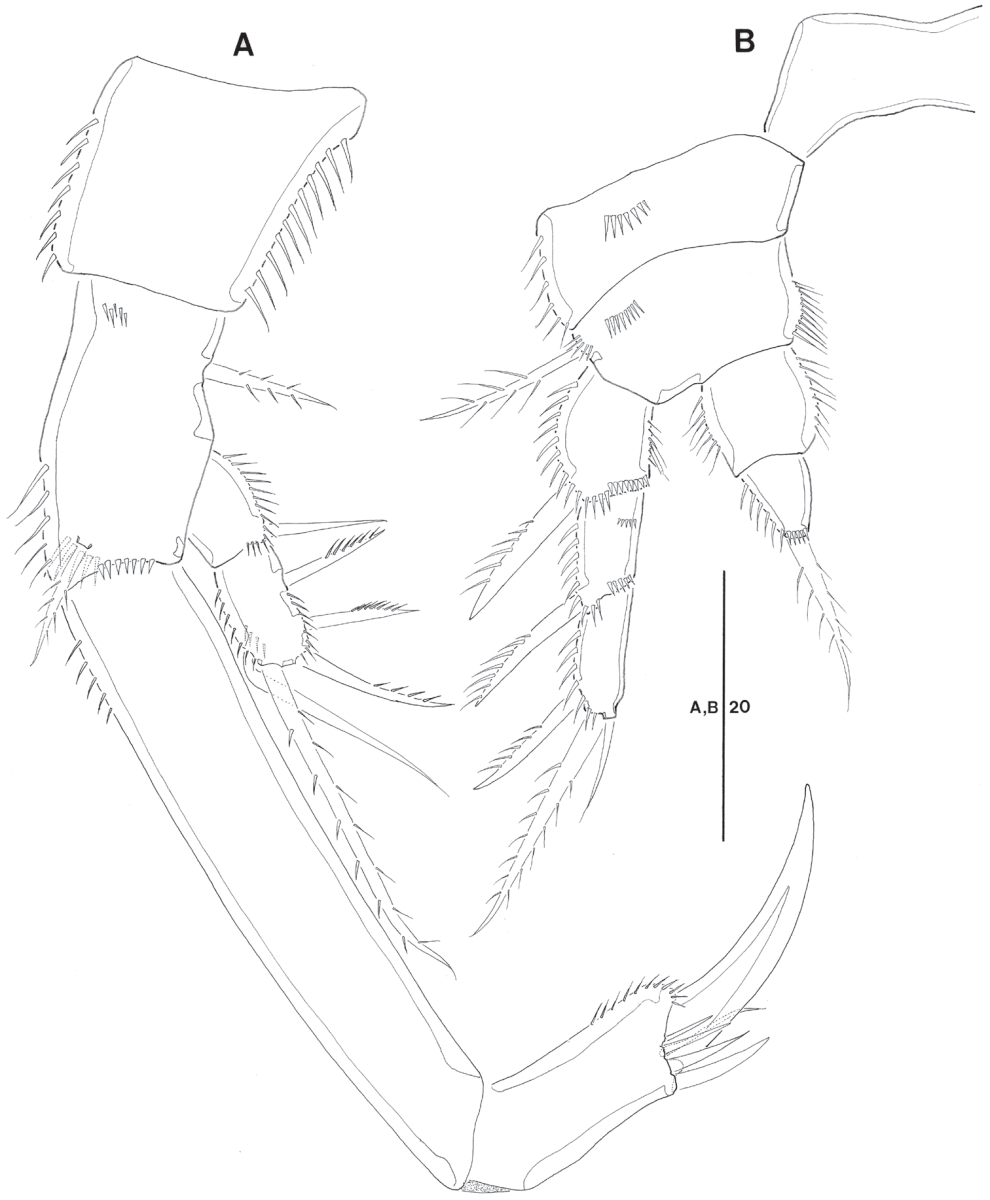
*Jejulaophonte hyeopjaeensis* gen. n., sp. n. (♀). **A** P1, anterior **B** P2, anterior.

P1 (Fig. 4A). Coxa ornamented with inner and outer spinules. Basis armed with 1 outer and 1 inner plumose setae. Exopod 2-segmented; exp-1 with 1 outer seta; exp-2 equal in length of exp-1, with 5 setae. Endopod 2-segmented; enp-1 over 3 times longer than exopod, longitudinal coarse spinules proximally; enp-2 (Fig. 8C) with 1 small accessory seta, 1 large strong claw, and ornamented with 4 big spinules arranged around distal inner margin.

P2 (Fig. 4B). Coxa with dense ornamentation on anterior surface and along outer margin. Basis with 1 outer plumose seta near outer distal corner. Exopod 3-segmented, about 2 times longer than endopod; exp-1 ornamented with spinules along lateral and anterior margin, setules along inner margin and 1 pinnate spine; exp-2 with 1 stout pinnate spine and spinules along outer margin; exp-3 with 3 elements. Endopod 2-segmented, enp-1 larger than enp-2; enp-1 without seta; enp-2 with 1 distal plumose seta.

**Figure 5.**
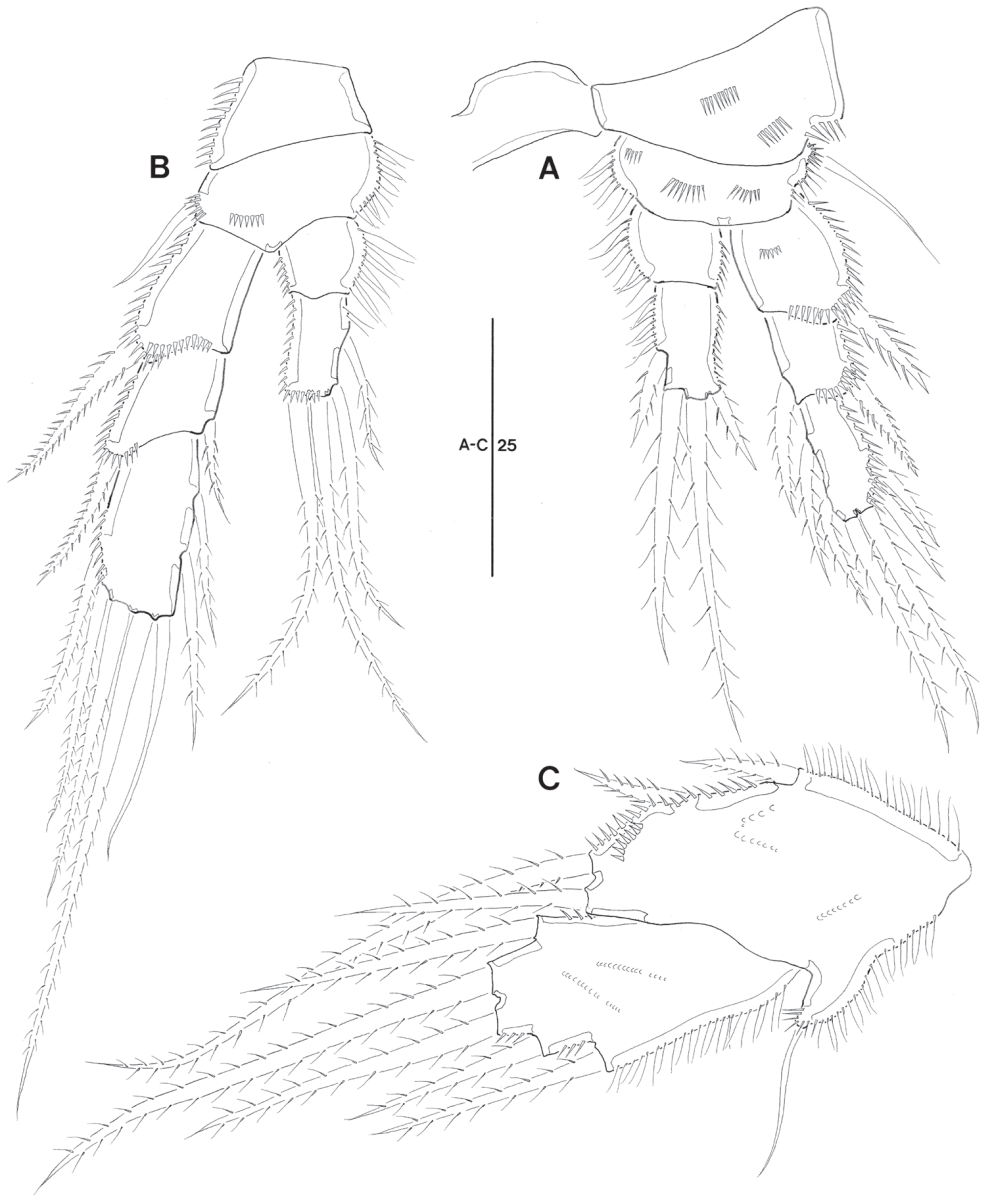
*Jejulaophonte hyeopjaeensis* gen. n., sp. n. (♀). **A** P3, anterior **B** P4, anterior **C** P5, anterior.

P3 (Fig. 5A). Coxa with dense ornamentation on surface and along outer margin. Basis with 1 outer naked seta and ornamented with row of spinules on middle surface and along inner margin. Exopod 3-segmented; exp-1 armed with 1 strong outer spine; exp-2 with 1 inner and 1 outer setae; exp-3 with 2 inner, 2 distal, and 2 outer setae. Endopod 2-segmented, each segment furnished with a row of spinules on outer margin and long setules along inner margin; enp-1 without seta; enp-2 with 1 inner, 2 distal, and 1 outer setae.

P4 (Fig. 5B). Coxa with a row of spinules along outer margin. Basis with 1 naked seta. Exopod 3-segmented, 2 times longer than endopod; exp-1 with 1 outer spine; exp-2 with 1 outer spine and 1 inner seta; exp-3 with 2 inner, 3 distal, and 2 outer setae. Endopod 2-segmented; enp-1 bare, ornamented with long setules on inner margin; enp-2 with 4 setae.

Armature formulae as follows:

**Armature formulae:** 

	Exopod	Endopod
P2	0.0.021	0.010
P3	0.1.222	0.121 (0.0.111 in ♂)
P4	0.1.232 (0.1.231 in ♂)	0.121

P5 (Fig. 5C). Baseoendopod ornamented with long setules along inner and outer margins and 1 basal naked seta. Endopodal lobe small, with 4 pinnate setae. Exopod oblong, with 5 plumose setae, a row of short spinules on outer margin and long setules along inner margin.

##### Description of male.

**Figure 6.**
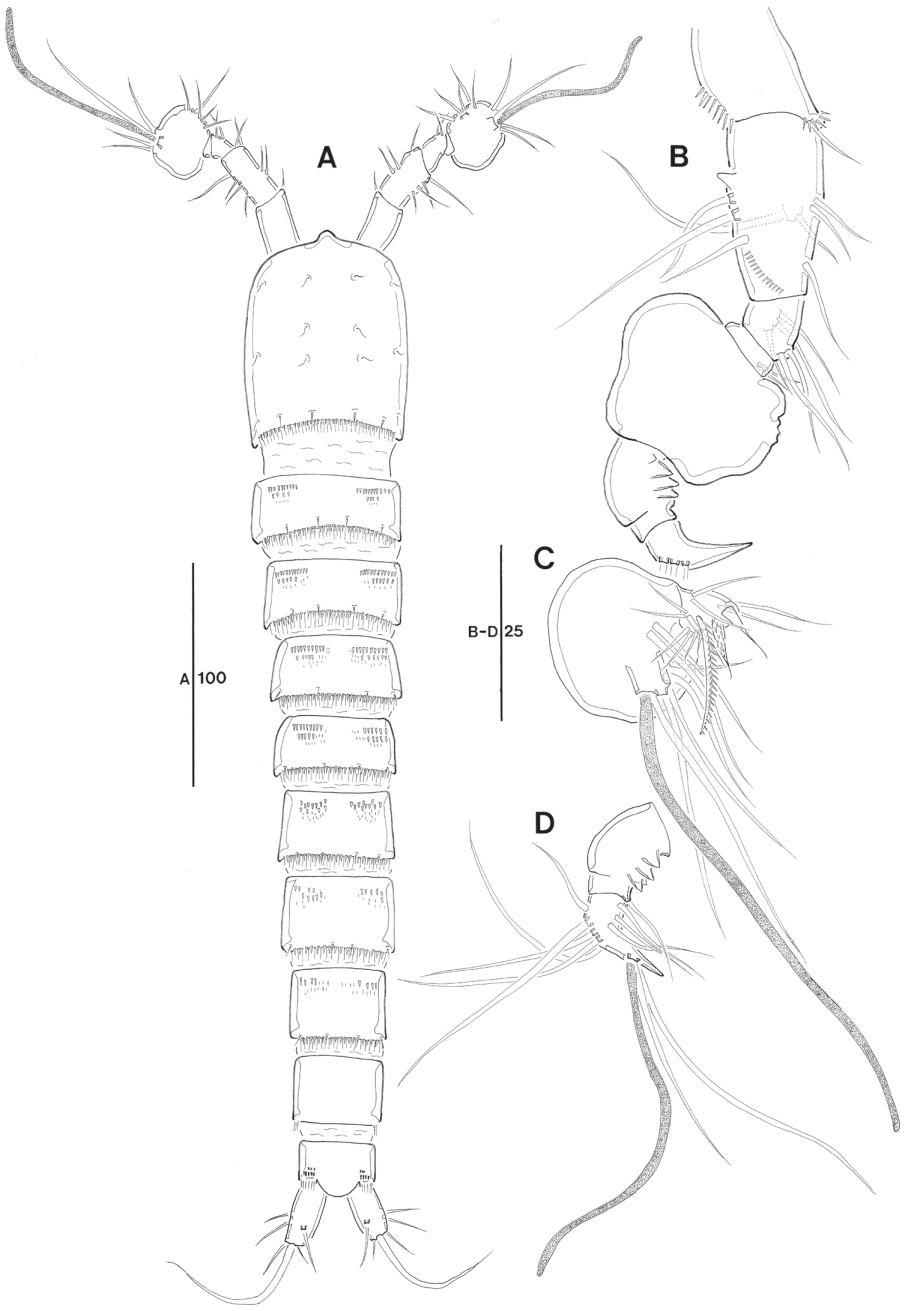
*Jejulaophonte hyeopjaeensis* gen. n., sp. n. (♂). **A** habitus, dorsal **B** antennule **C** antennulary segment 5 **D** antennulary segments 6 and 7.

Body (Fig. 6A) cylindrical, more compact than in female; length 450 µm (n = 4, mean = 451 μm) measured from anterior margin of cephalic shield to posterior margin of caudal ramus. Maximum width 76 µm at posterior margin of cephalothorax.

General body shape, ornamentation and sensilla pattern as in female. Sexual dimorphisms in A1, P3, P4, P5, and P6.

Antennule (Figs 6B–D, 8E). 7-segmented, robust, subchirocer. Segment-1 with 1 small seta on posterior margin. Segment-2 longest, with 1 small projection and 8 bare setae. Segment-5 with a proximal process anteriorly, and swollen. Segment-6 formed by 2 incompletely fused segments with 1 seta and 4 processes at inner margin. Distal position of segment-7 pointed, subtriangular, displacing acrothek to position isolated from other armature. Armature formula; 1–[1 bare], 2–[8 bare], 3–[5 bare], 4–[2 bare], 5–[17 bare + 1 modified + 1 pinnate + (1+ae)], 6–[1 + 4 processes], 7–[8 + acrothek]. Apical acrothek consisting of an aesthetasc and two naked setae.

Swimming legs 1–2 similar to those of female.

**Figure 7.**
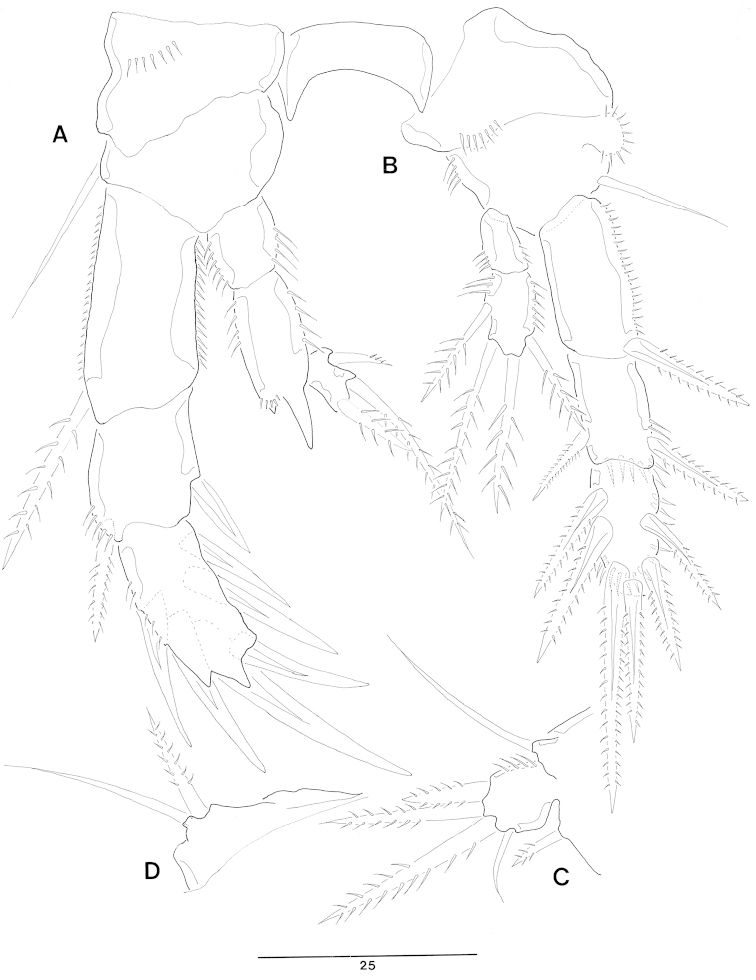
*Jejulaophonte hyeopjaeensis* gen. n., sp. n. (♂). **A** P3, anterior **B** P4, anterior **C** P5, anterior **D** P6, anterior.

**Figure 8.**
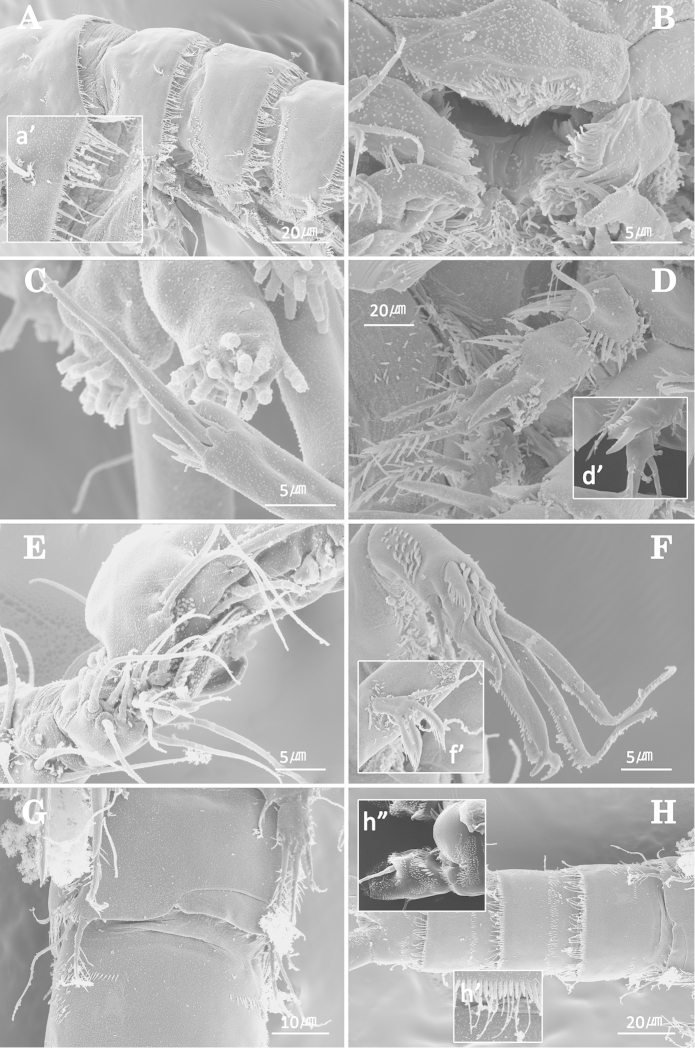
*Jejulaophonte hyeopjaeensis* gen. n., sp. n. **A** prosome in ♀, lateral, a’, dorso-posterior margin of prosomites **B** mouthparts part in ♀ **C** distal part of P1 endopod-2 in ♀ **D** apophysis of P3 endopod in ♂, d’; small processes on distal outer corner of P3 endopod-2 **E** antennulary segments 3, 4 and 5 in ♂ **F** A2 endopod in ♂, f’ antennary exopod **G** P6 in ♂ **H** urosome, ventral, h’, ventro-posterior margin of urosomite, h’’, anal somite in ♂, dorsal.

P3 (Figs 7A, 8D). Coxa with dense ornamentation on anterior surface. Basis with 1 naked outer seta. Exopod 3-segmented, more robust than in female; exp-1 ornamented with a row of spinules along inner and outer margin with 1 modified outer spine longer than female; exp-2 the shortest, with 1 outer, and 1 inner spine; exp-3 with 6 modified stout spines. Endopod 3-segmented; enp-1 ornamented a row of spinules on outer margin and long setules along inner margin, without seta; enp-2 with distal inner corner produced as an apophysis without ornamentation and 4 small processes on distal outer corner (Fig. 8D); enp-3 shortest, located next to apophysis of exp-2 with 1 inner and 2 distal setae.

P4 (Fig. 7B). Coxa with dense ornamentation on posterior surface. Basis with 1 naked outer seta. Exopod 3-segmented, exp-1 and exp-2 with modified outer spines; exp-3 with 6 modified spines. Endopod 2-segmented, enp-1 without seta; enp-2 with 1 outer, 2 apical, and 1 inner setae, all setae pinnate.

P5 (Fig. 7C). Fused medially; baseoendopod fig flattened, defined at the base. Baseoendopod with 1 outer basal seta, and endopodal lobe represented by 1 bare seta. Exopod small, with 1 bare, and 3 pinnate setae.

P6 (Fig. 7D, 8G). Symmetrical, represented on both sides by a small fig(fused to ventral wall of supporting somite along one side; articulating at base and covering gonopore along one side); outer distal corner produced into a cylindrical process with 1 inner pinnate and 1 outer naked setae.

## Discussion

The new species has a unique character set including the seta formula of P2–P4, and the shape of P5. Based on the Wells’ key (2007), there is no extant genus that can harbor the specific character combination of the present new species. Especially, within the family Laophontidae the new species has a unique combination of three setae on P2 exp-3 and only one seta on P2 enp-2. The new genus, *Jejulaophonte* is placed in the subfamily Laophontinae based on character sets including the sub-chirocer male antennule, the typically uniramous mandible, the syncoxa of maxilliped armed with maximum only two setae, the P1 enp-1 without inner seta, the reduced P2 enp-2 without outer spine, and the proximal outer setae of female P5 exopod with a distinctly separated insertion site. *Jejulaophonte* is closely related to five genera (*Carraroenia* McCormack, 2006, *Coullia* Hamond, 1973, *Hemilaophonte* Jakubisiak 1933, *Psammoplatypus* Lee & Huys, 1999, and *Robustunguis* Fiers, 1992), the CCHPR-lineage, based on the reduced P2 endopod (Laophontidae typically has P2 larger than P3), the ovate shape of female P5 exopod, and the sexual dimorphism in the P3 endopod (Gomez and Boyko 2006, Huys 2009, McCormack 2006). Fiers (1992a, b) claimed that the main reason for some species of the genera *Robustunguis* and *Hemilaophonte* having reduced appendages is to adapt to their host. Lee and Huys (1999) discussed the relationship between *Psammoplatypus* and related genera based on the reduced P2 endopod, the swimming leg sexual dimorphism and the ovate shape of female P5 exopod. Additionally, McCormack (2006) observed that the species *Carraroenia ruthae* McCormack, 2006 shares some characters with this lineage. Huys (2009) consequently suggested that *Phycolaophonte* and *Eolaophonte* should be subsumed into the synonymy of *Coullia* and provided a key to genera which have reduced P2 including the five genera *Carraroenia*, *Coullia*, *Hemilaophonte*, *Psammoplatypus* and *Robustunguis*. Especially, he recognized the reduced P2 endopod, sexual dimorphism in P3 endopod of male, and ovate shape of P5 as the shared characters. Importantly, the new genus shares those characters with the CCHPR-lineages.

While the new genus shares the reduced P2 endopod with the CCHPR-lineage, there are several conspicuous differences in the seta formula of appendages (Table 1). *Jejulaophonte*, *Psammoplatypus*, and *Carraroenia* differ from its congeners in the presence of an inner seta on P3–P4 exp-2, four setae on P4 enp-2 (instead of 2 or 3 setae in other genera), one inner seta on P3–P4 enp-2 (instead of 0) in the female. On the other hand, *Jejulaophonte* shares the primitive characters of five setae on P1 exp-2 with *Coullia insularis* (Pallares, 1975), *Coullia tongariki* (Gomez & Boyko, 2006), and *Psammoplatypus discipes* (Noodt, 1958), one or two inner setae on P4 exp-3 with *Coullia insularis*, and *Coullia tongariki* (Table 1). However, *Carraroenia* can be regarded as the most primitive genus in the lineage rather than *Jejulaophonte* and *Psammoplatypus*, by having two inner setae in P4 enp-2, the retention of a inner seta on P2 exp-2 and the primitive P5 armed with six setae on exopod and with five setae on baseoendopod (Mccormack 2006). According to Huys’ (2009) key, the new genus belongs to the group with *Carraroenia* and *Pasmmoplatypus* by having an inner seta on P4 exp-2. The genus *Psammoplatypus* can be regarded as the closest sister group of *Jejulaophonte*, new genus, because of the absence of inner seta of P2 exp-2.

**Table 1.** Armature formulae of five genera in the CCHPR-lineage related to the new genus, *Jejulaophonte*.

Genus	species	A1	P1	P2		P3		P4		P5	
			exp-2	exp	enp	exp	enp	exp	enp	exp	benp
*Robustunguis*	*ungulatus* (♀)	6	4	0.0.022	010	0.0.022	020	0.0.022	020	6	4
	(♂)	6	4	0.1.022	0.010	0.0.022	0.020	0.0.022	0.021	5	2
	*minor* (♀)	6	4	0.022	010	0.022	0.020	0.022	1	5	0
	(♂)	6	4	0.022	010	0.022	0.020	0.022	1	1	?
*Carraroenia*	*ruthae* (♀)	6	4	0.1.023	0.010	0.1.123	0.121	0.1.123	0.220	6	5
	(♂)	8	4	0.1.023	0.010	0.1.123	0.0.020	0.1.123	0.111	5	2
*Psammoplatypus*	*discipes* (♀)	6	5	0.0.023	0.020	0.1.023	0.121	0.1.023	0.121	4	5
	(♂)	6	4	-	0.120	-	0.0.120	-	-	4	2
	*proprius* (♂)	8	4	0.0.023	0.120	0.1.023	0.0.020	0.1.023	0.120	4	2
*Hemilaophonte*	*janinae* (♀)^1^	6	4	0.0.023	0.020	0.0.023	0.020	0.022	0.021	6	4
	*janinae* (♂)^1^	6		0.0.023	0.020	0.0.023	0.020	0.022	0.021	5	2
	*janinae* (♀)^2^	6	4	0.0.022	020	-	-	0.022	0.011	6	3
	*janinae* (♂)^2^	-	-	-	-	-	-	-	-	4	3
*Coullia*^3^	*clysmae* (♀)	6	4	0.0.023	absent	0.0.023	021	0.0.022	021	6	4
	*heteropus* (♀)	6	4	0.0.023	010	0.0.023	021	0.0.022	020	6	3
	*platychelipusoides* (♀)	7	4	0.0.023	0.020	0.0.023	0.021	0.0.022	0.011	6	5
	*mediterranea* (♀)	?	4	0.0.023	0.020	0.0.023	0.010	0.0.022	0.021	6	4
	*insularis* (♀)	6	5	0.0.023	020	0.0.023	0.021	0.0.023	0.021	6	5
	(♂)	?	5	0.1.023	0.020	0.1.023	0.020	0.0.022	0.021	5	2
	*tongariki* (♀)	6	5	0.1.023	0.020	0.0.023	0.021	0.0.023	0.021	6	5
*Jejulaophonte*	*hyeopjaeensis* (♀)	6	5	0.0.021	0.010	0.1.222	0.121	0.1.232	0.121	5	4
	(♂)	7	5	0.0.021	0.010	0.1.222	0.0.111	0.1.231	0.121	4	1

^1^ Based on Fiers 1992b.

^2^ Based on Jakubisiak 1932 [put in the Literature cited].

^3^ Based on Huys 2009.

### An updated key including *Jejulaophonte* is as follows (amended from Huys 2009)

**Table:** 

1	P1 well developed; longer than half of body length; P2 endopod 1-segmented; distal segment of P3 exopod with 4 elements in both sexes	***Robustunguis***
–	P1 shorter than half of body, these characters not combined	**2**
2	P4 exp-2 with inner seta in both sexes; P4 enp-2 with 4 elements in female	**3**
–	P4 exp-2 without inner seta; P4 enp-2 with 3 elements at most in female	**5**
3	P2 exp-2 with inner seta	***Carraroenia***
–	P2 exp-2 without inner seta	**4**
4	P2 and P3 exp-3 with 5 elements in both sexes	***Psammoplatypus***
–	P2 exp-3 with 3 elements and P3 exp-3 with 6 elements in both sexes	***Jejulaophonte* gen. n.**
5	P4 exopod 2-segmented	***Hemilaophonte***
–	P4 exopod 3-segmented	***Coullia***

Although the female of *Psammoplatypus proprius* (Lang, 1965) has not yet been described, we suppose that the seta formula in the distal segment of P2 and P3 exopod is common in both sexes as the other species in this group do not have sexual dimorphism in the seta formula on the distal segment of P2 and P3 exopod (Table 1).

**Table 2.** List of harpacticoid species with mt COI gene in the GenBank.

Family	Genus	species	Reference
Canthocamptidae	*Australocamptus*	*hamondi*	Karanovic and Cooper 2011b
	*Cletocamptus*	*deitersi*	Rocha-Oliveres et al. 2001
	*Cletocamptus*	*helobius*	Rocha-Oliveres et al. 2001
	*Elaphoidella*	*humphreysi*	Karanovic and Cooper 2011b
Darcythompsoniidae	*Leptocaris*	*canariensis*	Unpublished
Harpacticidae	*Tigriopus*	*brevicornis*	Jung et al. 2006
	*Tigriopus*	californicus*	Burton 1998
	*Tigriopus*	*fulvus*	Edmands 2001
	*Tigriopus*	japonicus*	Peterson et al. 2013
Laophontidae	*Jejulaophonte*	*hyeopjaeensis*	This study
Miraciidae	*Macrosetella*	*gracilis*	Eberl et al. 2007
	*Miracia*	*efferata*	Bucklin et al. 2010
	*Schizopera*	*akation*	Karanovic and Cooper 2012
	*Schizopera*	*akolos*	Karanovic and Cooper 2012
	*Schizopera*	*analspinulosa*	Karanovic and Cooper 2012
	*Schizopera*	*analspinulosa linel*	Karanovic and Cooper 2012
	*Schizopera*	cf. *uranusi*	Karanovic and Cooper 2012
	*Schizopera*	*emphysema*	Karanovic and Cooper 2012
	*Schizopera*	*kronosi*	Karanovic and Cooper 2012
	*Schizopera*	*leptafurca*	Karanovic and Cooper 2012
	*Schizopera*	*uranusi*	Karanovic and Cooper 2012
Paramesochridae	*Remanea*	*naksanensis*	Back et al. 2011
Parastenocarididae	*Dussartstenocaris*	*idioxenos*	Karanovic and Cooper 2011b
	*Kinnecaris*	*lined*	Karanovic and Cooper 2011a
	*Kinnecaris*	*linel*	Karanovic and Cooper 2011b
	*Kinnecaris*	*linesae*	Karanovic and Cooper 2011a
	*Kinnecaris*	*uranusi*	Karanovic and Cooper 2011b
	*Parastenocaris*	*jane*	Karanovic and Cooper 2011b

* There are many references of cytochrome c oxidase subunit I (mt COI) on NCBI, and a recent data was selected for the table.

While *Jejulaophonte* shares some primitive characters with the lineage, the new species can be distinguished from the species of CCHPR-lineage by the reduced P5 setation. Except for *Robustunguis minor* Fiers, 1992 and *Psammoplatypus discipes*, all species in the CCHPR-lineage share the characters of the reduced P2, and the six setae on the P5 exopod in the female. However, the new species possesses a reduced setation of five setae on the P5 exopod in the female and four setae in the male. Furthermore, endopodal lobe of the male has only one seta (the others of CCHPR-lineage have at least two setae except for *Robustunguis minor*).

The nuclear ribosomal genes are useful for phylogenetic study (Huys et al. 2006, Huys 2009), however the mitochondrial cytochrome c oxidase subunit I (mtCOI) gene was proposed as a ‘barcode’ (Hebert et al. 2003; Bradford et al. 2010). Until now, mtCOI sequences of 27 harpacticoid species including the new species and the unpublished species *Leptocaris canariensis* were updated on GenBank (Table 2). The sequences of the new species are the first barcode in the family Laophontidae, and it would be a useful temfig for laophontid barcode study.

## Supplementary Material

XML Treatment for Jejulaophonte

XML Treatment for Jejulaophonte hyeopjaeensis

## References

[B1] Boxshall GA Halsey SH (2004) An introduction to copepod diversity. The Ray Society, London, 2000 pp

[B2] Back J Lee W Huys R (2011) A new species of *Remanea* Klie, 1929 (Copepoda; Harpacticoida; Paramesochridae) with a redescription of the type species. Journal of Natural History 45: 2939–2964. doi: 10.1080/00222933.2011.622057

[B3] Bradford T Adams M Humphreys F Austin AD Cooper SJB (2010) DNA barcoding of stygofauna uncovers cryptic amphipod diversity in a calcrete aquifer in Western Australia’s arid zone. Molecular Ecology Resources 10: 41–50. doi: 10.1111/j.1755-0998.2009.02706.x 21564989 10.1111/j.1755-0998.2009.02706.x

[B4] Bucklin A Ortman BD Jennings RM Nigro LM Sweetman CJ Copley NJ Sutton T Wiebe PH (2010) A “Rosetta Stone” for metazoan zooplankton: DNA barcode analysis of species diversity of the Sargasso Sea (Northwest Atlantic Ocean). Deep Sea Research II 57: 2234–2247. doi: 10.1016/j.dsr2.2010.09.025

[B5] Burgess R (2001) An improved protocol for separation meiofauna from sediments using colloidal silica sols. Marine Ecology Progress Series 214: 161–165. doi: 10.3354/meps214161

[B6] Burton RS (1998) Intraspecific Phylogeography Across the Point Conception Biogeographic Boundary. Evolution 52: 734–745. doi: 10.2307/2411268 10.1111/j.1558-5646.1998.tb03698.x 28565261

[B7] Eberl R Cohen S Cipriano F Carpenter EJ (2007) Genetic diversity of the pelagic harpacticoid copepod *Macrosetella gracilis* on colonies of the cyanobacterium *Trichodesmium* spp. Aquatic Biology 1: 33–43. doi: 10.3354/ab00002

[B8] Edmands S (2001) Phylogeography of the intertidal copepod *Tigriopus californicus* reveals substantially reduced population differentiation at northern latitudes. Molecular Ecology 10: 1743–1750. doi: 10.1046/j.0962-1083.2001.01306.x 11472541 10.1046/j.0962-1083.2001.01306.x

[B9] Fiers F (1992a) A redescription of *Hemilaophonte janinae* Jakubisiak (Copepoda, Harpacticoida), a laophontid living in the gill chambers of the common spider crab. Belgian Journal of Zoology 122(2): 211–222

[B10] Fiers F (1992b) *Robustunguis* gen. n., a genus of decapod associated laophontids (Copepoda: Harpacticoida). Zoologische Mededelingen Leiden 66(28): 399–412

[B11] Folmer O Black M Hoen W Lutz R Vrijenhoek R (1994) DNA primers for amplication of mitochondrial cytochrome c oxidase subunit I from diverse metazoan invertebrates. Molecular Marine Biology and Biotechnology and Biotechnology 3: 294–299 7881515

[B12] Gheerardyn H Fiers F Vincx M De Troch M (2007) *Spiniferaphonte*, a new genus of Laophontidae (Copepoda: Harpacticoida), with notes on the occurrence of processes on the caudal rami. Journal of Crustacean Biology 27: 309–318. doi: 10.1651/S-2723.1

[B13] Gomez S Boyko CB (2006) On a small collection of harpacticoids from Easter Island: the family Laophontidae T. Scott (Crustacea: Copepoda: Harpacticoida). Zootaxa 1352: 3–70

[B14] Hebert PDN Cywinska A Ball SL de Waard JR (2003) Biological identifications through DNA barcodes. Proceeding of the Royal Society of London Series B Biological Science 270: 313–321. doi: 10.1098/rspb.2002.2218 10.1098/rspb.2002.2218 PMC1691236 12614582

[B15] Hicks GRF (1988) Systematics of the Donsiellinae Lang (Copepoda, Harpacticoida). Journal of Natural History 22: 639–684. doi: 10.1080/00222938800770441

[B16] Huys R (2009) On the junior subjective synonyms of *Coullia* Hamond, 1973 (Copepoda, Harpacticoida, Laophontidae): an update and key to species and related genera. Zookeys 5: 33–40. doi: 10.3897/zookeys.5.64

[B17] Huys R Gee JM Moore CG Hamond R (1996) Marine and Brackish Water Harpacticoid Copepods Part 1. Synopses of the British Fauna (New Series) 51: 1–352

[B18] Huys R Lee W (2000) Basal resolution of laophontid phylogeny and the paraphyly of *Esola* Edwards. Bulletin of the Natural History Museum. Zoology Series 66: 49–107

[B19] Huys R Llewellyn–Hughes J Olson PD Nagasawa K (2006) Small subunit rDNA and Bayesian inference reveal *Pectenophilus ornatus* (Copepoda incertae sedis) as highly transformed Mytilicolidae, and support assignment of Chondracanthidae and Xarifiidae to Lichomolgoidea (Cyclopoida). Biological Journal of the Linnean Society 87: 403–425. doi: 10.1111/j.1095-8312.2005.00579.x

[B20] Huys R Willems KA (1989) *Laophontopsis* Sars and the taxonomic concept of the Normanellinae (Copepoda: Harpacticoida): A revision. Bijdragen tot de Dierkunde 59: 203–227

[B21] Jakubisiak S (1932) Sur les Harpacticoïdes hébergés par Maia squinado. Bulletin de la Société zoologique de France 57: 506–513

[B22] Jung SO Lee YM Park TJ Park HG Hagiwara A Leung KMY Dahms HU Lee W Lee JS (2006) The complete mitochondrial genome of the intertidal copepod *Tigriopus* sp. (Copepoda, Harpactidae) from Korea and phylogenetic considerations. Journal of Experimental Marine Biology and Ecology 333: 251–262. doi: 10.1016/j.jembe.2005.12.047

[B23] Karanovic T Cooper SJB (2011a) Molecular and morphological evidence for short range endemism in the *Kinnecaris solitaria* complex (Copepoda, Parastenocarididae), with descriptions of seven new species. Zootaxa 3026: 1–64

[B24] Karanovic T Cooper SJB (2011b) Third genus of parastenocarid copepods from Australia supported by molecular evidence (Harpacticoida: Parastenocarididae). In: Studies on Freshwater Copepoda: a Volume in Honour of Bernard Dussart. Crustaceana Monographs 16: 283–326

[B25] Karanovic T Cooper SJB (2012) Explosive radiation of the genus *Schizopera* Sars (Copepoda: Harpacticoida) in a small subterranean island in Western Australia: unravelling the cases of cryptic speciation, size differentiation, and multiple invasions. Invertebrate Systematics 26: 115–192. doi: 10.1071/IS11027

[B26] Lang K (1944) Monographie der Harpacticiden (Vorläufige Mitteilung). Almqvist & Wiksells, Uppsala, 39 pp

[B27] Lang K (1965) Copepoda Harpacticoidea from the Californian Pacific coast. Kunglieren svenska Vetenskapsakademiens Handlingar 10: 1–560

[B28] Lee W Huys R (1999) *Bathylaophonte* gen. n. from deep-sea hydrothermal vents and the polyphyly of *Paronychocamptus* (Copepoda: Harpacticoida). Cahiers de Biologie Marine 40: 293–328

[B29] McCormack E (2006) *Carraroenia ruthae* gen. et sp. nov. (Copepoda, Harpacticoida, Laophontidae) from maerl substrates of the Irish west coast. Zootaxa 1202: 39–52

[B30] Peterson DL Kubow KB Connolly MJ Kaplan LR Wetkowski MM Leong W Phillips BC Edmand S (2013) Reproductive and phylogenetic divergence of tidepool copepod populations across a narrow geographical boundary in Baja California. Journal of Biogeography 40: 1664–1675. doi: 10.1111/jbi.12107

[B31] Philippi A (1840) Zoologische Bemerkungen (Fortsetzung). IV. Kurze Charakteristik mehrerer neuer Genera aus der Familie der Copepoden. Archiv für Naturgeschichte 6: 188–190

[B32] Rocha-Olivares A Fleeger JW Foltz DW (2001) Decoupling of molecular and morphological evolution in deep lineages of a meiobenthic harpacticoid copepod. Molecular Biology and Evolution 18: 1088–1102. doi: 10.1093/oxfordjournals.molbev.a003880 11371597 10.1093/oxfordjournals.molbev.a003880

[B33] Scott T (1905) On some new and rare Crustacea from the Scottish seas. Report of the Fishery Board for Scotland 23: 141–153

[B34] Wells JBJ (2007) An annotated checklist and keys to the species of Copepoda Harpacticoida (Crustacea). Zootaxa 1568: 1–872

[B35] Willen E (1996) Two new genera of Laophontidae (Copepoda: Harpacticoida) from the high Antarctic Weddell Sea. Journal of Natural History 30: 1297–1327. doi: 10.1080/00222939600771231

